# Investigating COVID-19 Vaccine Communication and Misinformation on TikTok: Cross-sectional Study

**DOI:** 10.2196/38316

**Published:** 2022-10-25

**Authors:** Katherine van Kampen, Jeremi Laski, Gabrielle Herman, Teresa M Chan

**Affiliations:** 1 Michael G DeGroote School of Medicine Faculty of Health Sciences McMaster University Hamilton, ON Canada; 2 College of Medicine Central Michigan University Mount Pleasant, MI United States; 3 Division of Emergency Medicine, Department of Medicine Faculty of Health Sciences McMaster University Hamilton, ON Canada; 4 Office of Continuing Professional Development Faculty of Health Sciences McMaster University Hamilton, ON Canada; 5 McMaster Education Research, Innovation, and Theory Faculty of Health Sciences McMaster University Hamilton, ON Canada; 6 Division of Education & Innovation Department of Medicine, Faculty of Health Sciences McMaster University Hamilton, ON Canada

**Keywords:** TikTok, COVID-19 vaccines, vaccinations, misinformation, COVID-19, Infodemiology, social media, health information, content analysis, vaccine hesitancy, public health, web-based health information

## Abstract

**Background:**

The COVID-19 pandemic has highlighted the need for reliable information, especially around vaccines. Vaccine hesitancy is a growing concern and a great threat to broader public health. The prevalence of social media within our daily lives emphasizes the importance of accurately analyzing how health information is being disseminated to the public. TikTok is of particular interest, as it is an emerging social media platform that young adults may be increasingly using to access health information.

**Objective:**

The objective of this study was to examine and describe the content within the top 100 TikToks trending with the hashtag #covidvaccine.

**Methods:**

The top 250 most viewed TikToks with the hashtag #covidvaccine were batch downloaded on July 1, 2021, with their respective metadata. Each TikTok was subsequently viewed and encoded by 2 independent reviewers. Coding continued until 100 TikToks could be included based on language and content. Descriptive features were recorded including health care professional (HCP) status of creator, verification of HCP status, genre, and misinformation addressed. Primary inclusion criteria were any TikToks in English with discussion of a COVID-19 vaccine.

**Results:**

Of 102 videos included, the median number of plays was 1,700,000, with median shares of 9224 and 62,200 followers. Upon analysis, 14.7% (15/102) of TikToks included HCPs, of which 80% (12/102) could be verified via social media or regulatory body search; 100% (15/15) of HCP-created TikToks supported vaccine use, and overall, 81.3% (83/102) of all TikToks (created by either a layperson or an HCP) supported vaccine use.

**Conclusions:**

As the pandemic continues, vaccine hesitancy poses a threat to lifting restrictions, and discovering reasons for this hesitancy is important to public health measures. This study summarizes the discourse around vaccine use on TikTok. Importantly, it opens a frank discussion about the necessity to incorporate new social media platforms into medical education, so we might ensure our trainees are ready to engage with patients on novel platforms.

## Introduction

Social media has become a prominent vehicle for educating both learners and the public. Learners and young physicians are increasingly savvy with these technologies [1,2], engaging as influencers and gaining outsized influence over young people [3].

Although the rapid development and emergency approval of multiple vaccines is something to be celebrated, vaccine hesitancy and misinformation remain significant obstacles to global vaccination. Vaccine hesitancy has been noted as one of the greatest threats to global health by the World Health Organization in 2019 [4-6]. In particular, this is evident by lower vaccination rates in some countries such as the United States [4]. In comparison, other G7 countries have higher percentages of their citizens receiving at least one dose [7]. This misinformation may stem from social media use. A total of 82% of Americans use social media, and many may use it for health information [8]. Social media, including Twitter, Facebook, Instagram, and TikTok, among others, has fueled rumors, hoaxes, misinformation, as well as disinformation [9].

Misinformation occurs when incorrect information is unintentionally propagated [10]. Even more worryingly, the use of targeted disinformation, where medical facts are intentionally falsified, can propagate distrust of public health measures, such as mask wearing or vaccination [9,11]. Social media platforms have increasingly faced more pressure from both citizens and regulators alike to combat this disinformation [12]. Nevertheless, these platforms continue to be ongoing sources of both misinformation and disinformation, revealing a need to understand the vaccine discourse on these platforms [13]. A recent study by Griffith et al [14] explored some of the etiology of vaccine hesitancy by analyzing over 500 Twitter tweets containing COVID-19 vaccine hesitancy content. Several overarching themes related to vaccine hesitancy were identified that included concerns of safety, lack of knowledge about the vaccine, mistrust of the medical community, confusing messages from authority figures, and mistrust of vaccine companies [14].

TikTok is the twin of “Douyin”—the Chinese short video app, originally known as “Musical.ly”—later rebranded as TikTok to a western audience [15]. Founded in 2018, TikTok is a growing social media platform in which users upload short videos under 120 seconds. Users interact with the platform typically by the “For You” page, which is meant to algorithmically present videos in which the user may be interested [11,16]. Gaining incredible popularity, about 1 in 6 people in the United States are current TikTok users [17]. However, despite its popularity, TikTok’s algorithm has come under criticism for perpetuating misinformation. A report by NewsGuard [18] found that TikTok accounts that spread vaccination misinformation and antivaccination sentiments were being viewed by children as young as age 9, although the app technically only allows users over the age of 13 to use it. Prior to TikTok’s revision of their algorithm, interacting with a single video containing false medical information could modify the “For You” page to be populated with similarly oriented vaccine hesitancy and COVID-19 misinformation content [19].

Given the vast implications of perpetuating medical misinformation during the onset of the COVID-19 pandemic, past research has sought to explore TikTok’s role in vaccine misinformation. A study from the end of 2020 [3] found more TikToks overall that discouraged vaccination; however, those encouraging vaccination gained more traffic. TikToks pertaining to vaccination typically included humor or parody, with parodies of adverse reactions gaining higher view counts [3,11]. Furthermore, a small number of TikToks included health care professionals (HCPs), and a few TikToks conveyed medical education [11]. TikToks pertaining to vaccination seem to be created by a majority of non-HCP creators, and vaccine hesitancy prevails as a common theme on the platform. It is imperative that social media platforms be analyzed to reveal public attitudes toward vaccination and allow for more targeted public health campaigns [11,20].

To close the gap between public perceptions and the science behind vaccines, there is certainly an avenue for engaging learners and providing them with tools to engage with the public more robustly [21]. Instead of engaging in financial gain via social media stardom [1], increased efforts to formalize social media use and communication skills and incorporate them into medical school curricula may be of great benefit to our communities. However, to do so, it is imperative that we have a firm handle on what the current state of web-based communications are for physicians and other HCPs on platforms like TikTok.

Our cross-sectional study seeks to examine trends and attitudes toward COVID-19 vaccines by analyzing the most viewed TikToks with the hashtag #covidvaccine in July 2021 and specify which of these were generated by physicians and other HCPs.

## Methods

### Cross-sectional Study

We conducted a cross-sectional study of published TikToks with the hashtag #covidvaccine to characterize the discourse regarding vaccine use on the platform and to explore HCPs presence on the app regarding vaccine use. Furthermore, HCPs were identified and validated (ie, through regulatory bodies), which helped to show HPCs at what fields can better understand the sentiments of the general population, especially in regard to misinformation being spread.

### Data Extraction

The most viewed TikToks with the hashtag #covidvaccine were batch downloaded using the open source TikTokApi Python wrapper [22] on July 1, 2021, with their respective metadata (ie, number of views, likes, shares, comments, author followers, and hashtags; Multimedia Appendix 1). TikToks were subsequently reviewed by 2 authors (JL and KvK) and encoded or categorized deductively (Multimedia Appendix 2); discrepancies between the 2 reviewers were resolved by the third author (GH).

### Inclusion Criteria and Descriptive Coding of TikToks

**Figure 1:**
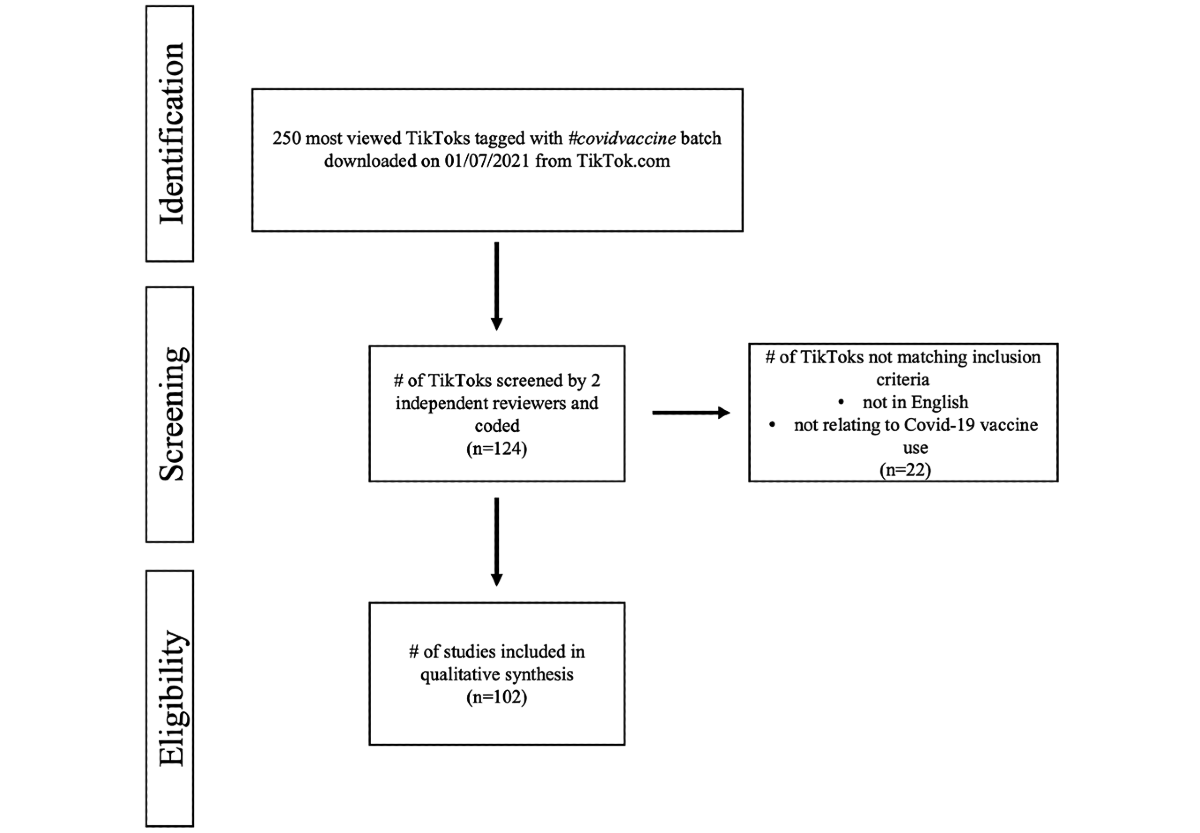
Schematic workflow with inclusion and exclusion criteria.

Primary inclusion criteria were any TikToks in English with discussion of a COVID-19 vaccine (either positive, negative, or neutral). Inclusion criteria were purposefully left as broad as possible to encompass as many TikToks that would refer to the COVID-19 vaccine and could be potentially viewed by a general TikTok user in the future. Exclusion criteria were TikToks in languages other than English and those not relating to COVID-19 vaccine or vaccine use (Figure 1). Review continued until we reached approximately 100 appropriate TikToks for analysis, for a total of 124 videos reviewed and 102 eligible. Descriptive features that were additionally recorded if possible included the following: number of people in the video, country of origin, HCP status of creator, verification of HCP status, type of medium (eg, dance, commentary, storytelling, question and answer, responding to comments, silent video with visuals, satire, skit, stitch, or other), scientific validity of claims evaluated at the time of TikTok creation, and if COVID-19 vaccine misinformation is either referred to or combatted. The agreement of the coded data (ie, whether to include or to exclude it) between the 2 reviewers (JL and KvK) was calculated (κ=0.64, 95% CI 0.63-0.64). Due to the ambiguous nature of the content and messaging of numerous TikToks, agreement of the coded data often required the input of the third reviewer, with consensus being reached following discussion between all 3 reviewers on whether the content was related to the COVID-19 vaccine. Indeed, in our preliminary investigation, we found that several TikToks met our broad inclusion criteria, but they used the hashtag #covidvaccine likely as a method of generating traffic to their TikTok, without actually mentioning any pertinent content related to vaccination.

### Data Visualization

The coding information was combined with the metadata to generate descriptive statistics and graphs. The data extraction and visualization workflow can be found in the study’s GitHub repository [23].

### Ethical Considerations

This study only analyzed publicly available data from existing data sets, and results do not contain any identifiable information that is not already in the public domain or are presented in aggregate.

## Results

### Overall Metrics

**Figure 2:**
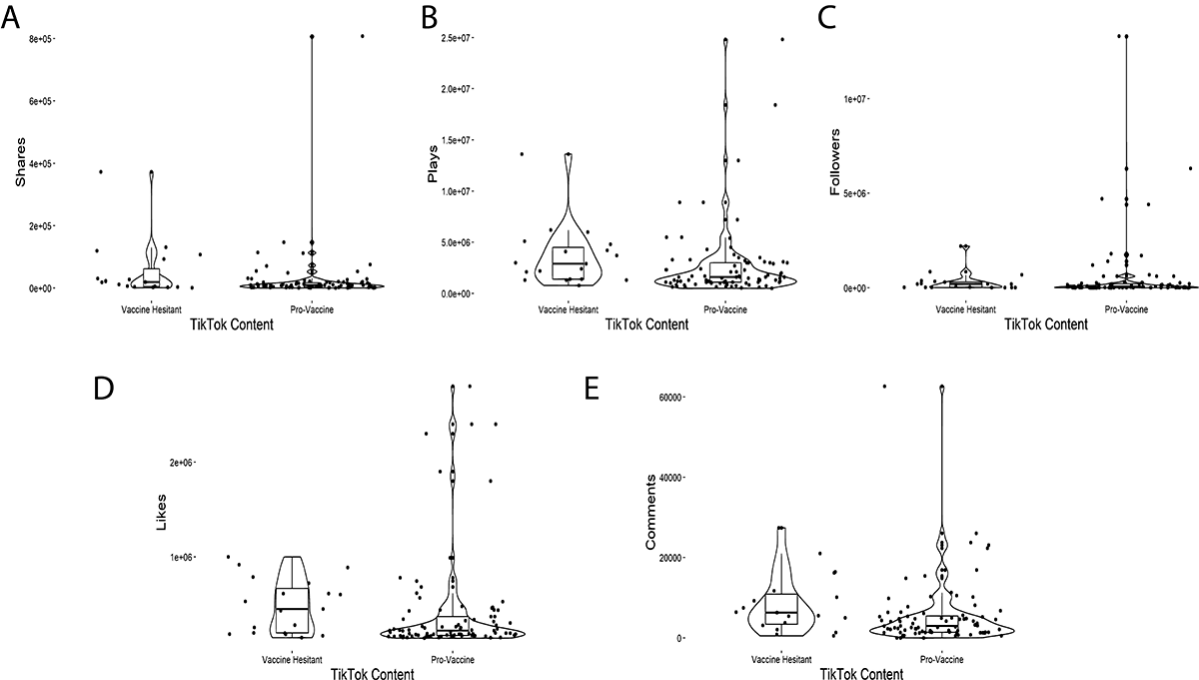
Violin plot depiction of individual TikTok metrics stratified as either vaccine hesitant (n=19) or pro-vaccine (n=83) as presented within Table 1.

**Figure 3:**
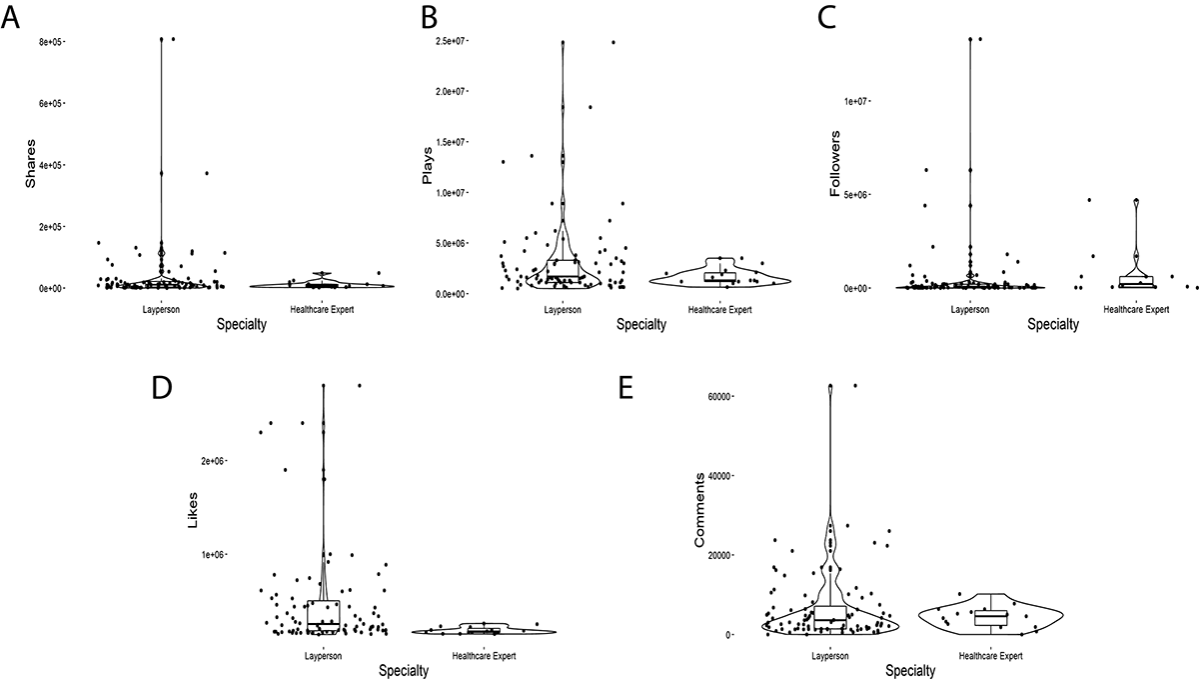
Violin plot depiction of individual TikTok metrics stratified by TikTok creator, either layperson (n=87) or health care expert (n=15), as presented within Table 2 (N=102).

Of the 102 coded TikToks, 19 (18.6%) contained vaccine-hesitant messaging, whereas 83 (81.3%) were provaccine. Median plays between these two groups were 290,000 and 160,000, respectively. Of note, many of the provaccine TikToks were simply people recording themselves receiving the vaccine or recording their experience and symptoms post vaccination. Other broad themes noted in the provaccine category were people celebrating vaccines as a measure to ending lockdowns, encouraging others to vaccinate themselves. Vaccine-hesitant TikToks generated higher median comments, shares, and author followers than provaccine TikToks (Table 1; Figure 2). Interestingly, a relatively low number (n=15, 14.7%) of total TikToks were attributed to HCP creators. These HCP TikToks, however, all contained provaccine content. Furthermore, when comparing TikToks created by either layperson or HCP creators, HCP TikToks had higher median plays, comments, shares, and author followers (Table 2; Figure 3). One particular HCP TikTok creator, Dr Noc, is of particular interest, as he is the only creator to have more than one (n=4) TikToks that fall into the top 102 TikTok category for the month of July 2021. We additionally investigated TikTok retention 4 months after our original analysis, on November 29, 2021. During this period, we assessed the number of TikToks that still remained on the internet and were viewable to the general public. Out of a total of 102 original TikToks, 94 (92.1%) still remained active. All removed TikToks (n=8, 7.9%) were from separate provaccine content creators.

**Table 1:** General metrics of characteristics for both vaccine-hesitant and provaccine individual TikToks.

Characteristics	Vaccine hesitant (N=19)^a^	Provaccine (N=83)^a^
Plays, median (IQR)	2,900,000 (1,400,000-4,500,000)	1,600,000 (1,100,000-3,000,000)
Likes, median (IQR)	447,800 (194,400-666,450)	220,600 (168,250-369,200)
Comments, median (IQR)	6253 (3402-10,900)	2963 (1408-5480)
Shares, median (IQR)	18,500 (4448-61,100)	8986 (2636-16,800)
Followers, median (IQR)	191,400 (17,150-312,150)	55,550 (7690-210,200)
Health care expert, n (%)	0 (0)	15 (18)
TikTok still present as of November 29, 2021, n (%)	19 (100)	75 (90)

^a^TikToks were categorized as created by either a layperson or health care expert.

**Table 2:** General metrics of individual TikTok characteristics created by laypeople or health care experts. Of note, 4 of the 15 health care expert–created TikToks are from the same user, Dr Noc.

Characteristics	Layperson (N=87)	Health care expert (N=15)
Supporting vaccine, n (%)	68 (78)	15 (100)
Plays, median (IQR)	1,700,000 (1,100,000-3,300,000)	1,300,000 (1,200,000-2,050,000)
Likes, median (IQR)	252,600 (180,800-501,900)	173,100 (162,250-205,100)
Comments, median (IQR)	3545 (1408-7108)	4562 (2268-5998)
Shares, median (IQR)	10,300 (3034-20,750)	6885 (3142-12,200)
Followers, median (IQR)	53,000 (7804-198,300)	209,000 (44,400-610,200)
TikTok still present as of November 29, 2021, n (%)	81 (93)	13 (87)

### Vaccine-Hesitant Content Misinformation Analysis

Beyond general TikTok metrics, we performed content analysis to identify certain perceptions and misinformation associated with TikToks (n=19, 18.6%) containing vaccine-hesitant content. Most of vaccine-hesitant TikToks (n=10, 53%) did not voice any particular vaccine-hesitant themes other than that the person chose not to get vaccinated. From the remaining (n=9, 47%) vaccine-hesitant TikToks, several vaccine-hesitant sentiments were noted as follows:

- We do not know the long-term side effects
- The vaccine injects you with a microchip
- The vaccine makes you magnetic

From these 9 vaccine-hesitant TikToks, 5 (55%) TikToks were listed as the individual creator’s “Top Liked” video; 8 of the 9 (88%) vaccine-hesitant TikToks pertained in some way to parodying or alluding to the vaccines causing neurological side effects that included dystonia or dysarthria; 2 out of the 9 (22%) vaccine-hesitant TikToks alleged that the vaccine injects you with a microchip that may make the individual magnetic.

### HCP Creator Verification

When attempting to verify HCP status of all (n=15) HCP-related TikToks, 12 (80%) Tik Toks were able to be attributed to a verified HCP through assessing medical professional registries, professional or academic institutions, and social media verification blue check marks (on TikTok and Instagram; Table 3). We were unable to verify 3 (20%) HCP-related TikToks, as either the creators purely self-reported HCP status or the TikTok account was deleted with no potential for follow-up investigation.

**Table 3:** Analysis of the specialty of the health care expert TikTok creators who were subsequently verified by JL and KvK. Total specialty number (n=12) does not align with total number of health care professional–created TikToks (n=15), as one creator within the research scientist category (Dr Noc) created multiple (n=3) TikToks within our analysis.

Specialty	Creators in each field, n	creators verified, n/N (%)
Physician	6	5/6 (83)
Nurse	2	2/2 (100)
Doula	1	0/1 (0)
Pharmacist	1	0/1 (0)
Phlebotomist	1	1/1 (100)
Research scientist (Immunology)	1	1/1 (100)

## Discussion

Our study characterized the content on TikTok during the summer of 2021 by analyzing TikToks tagged under the hashtag #covidvaccine. The results allow us to draw some conclusions regarding attitudes prevalent on TikTok during this time. Most of TikToks were supportive of vaccination, though the vaccine-hesitant content garnered more likes, shares, and views. HCPs represented a small portion of creators and all created provaccine content. Generally, vaccine-hesitant content reflected fears about side effects of the vaccine that were unfounded, such as magnetism.

Over 80% (n=83) of TikToks included in the study contained provaccine sentiments (Table 1). In comparison, Basch et al [3], who analyzed the same hashtag in March 2021, found only 36% of videos encouraging vaccination. As Basch et al did not code support for vaccines binarily (coding encompassed vaccine support, genre, and claims) [3], it is difficult to directly compare our study’s findings with their prior research. However, given the change over time, it’s suggestive that there was some increase in provaccine sentiments between the months of March and July 2021. This could be influenced by world events such as the increased distribution of vaccination around the globe. Between the months of March and July 2021, the number of individuals fully vaccinated against COVID-19 rose from 30.11 million to 159.79 million [24]. As vaccines became more available to the general population, more people may have posted about getting the vaccine [25]. In fact, many of our coded provaccine TikToks contained people recording themselves receiving their vaccination. It is also possible TikTok’s misinformation management algorithm may have changed their system for flagging and removing inaccurate videos. Currently, TikTok claims to combat medical misinformation by banning antivax advertisements, and it directs users to the World Health Organization’s website for COVID-19– related information [26]. They also claim to be removing TikToks containing misinformation within 24 hours [26]. TikTok USA promoted vaccine use on the platform, using #VaccinatedFor, a hashtag for users to share their reasons for being vaccinated [27]. Ultimately, the rise in provaccine TikToks is likely multifactorial. Some of the contributing factors may include the increasing proportion of vaccinated individuals, improved TikTok algorithm management for removal of misinformation, and increasing provaccine social outreach campaigns.

Nevertheless, even with the improvements in promoting vaccine use, none of the 9 TikToks containing misinformation from our original analysis were removed from the TikTok platform by November 2021. This suggests that TikTok may not be fully successful with their misinformation policy and is still struggling with detecting misinformation on the platform, with certain vaccine-hesitant content remaining on the app for more than 5 months. It is important to note that the deletion of provaccine content TikToks may have been due to a variety of reasons, such as the user leaving the TikTok app, or more worryingly, due to harassment over provaccine sentiments [28]. A possible reason for the great popularity of vaccine-hesitant TikToks may be the inclusion of misinformation that has a broad shock appeal [29]. We noted several TikToks that alluded to the vaccines’ side effect of making individuals magnetic. Although content on TikTok may generally be more provaccine, it cannot be ignored that the smaller portion of vaccine-hesitant videos gaining higher traffic represents a dangerous avenue for the spread of misinformation on TikTok.

Our analysis found that HCP-created TikToks accounted for 15% (n=15) of the total 102 most popular TikToks, and only 6% (n=6) were posted by physicians. This is only a slight increase from Southwick et al’s findings of 4% of individuals who were posting vaccine content on TikTok self-reporting as HCPs [11]. The small percentage of HCP-created popular TikToks suggests that HCPs can further use this platform to disseminate accurate medical information to a broad audience. However, with the advent of the medical influencer [1], it must be ensured that information provided by HCPs is correct and not biased by financial incentives. Although many current medical students have used social media for both their personal and professional lives, many have not received formalized social media training on how to disseminate information correctly beyond maintaining a professional image. Indeed, guidelines for maintaining a professional image have been created by both the American and Canadian Medical Associations [30,31]; however, these recommendations do not provide a guide on how to create new content that has educational value or helps combat misinformation. Social media platforms will continue to keep growing and gaining new followers, regardless of whether the health care community participates or not. As such, it is imperative that health care programs, residencies, and medical schools offer training to providers who choose to engage in medical education to broaden the reach of HCPs on social media.

### Limitations

As this was a cross-sectional study, there are inherent limitations to interpreting trends found on TikTok for the month of July. The TikToks deemed viral at the time may not be viral currently, and this may change the viewership metrics. Future studies may benefit from comparing several cross-sectional studies and perform content analysis on how trends change over time as more vaccinations are rolled out globally. Our data extraction is also limited by TikTok’s algorithm, which is known to show users content related to their interests. Although the extracted TikToks were highly viewed, it is difficult to determine whether the views originated from people being recommended content by the “For You” page algorithm or whether individuals specifically searched out the #covidvaccine hashtag. Due to the small sample size of HCP content creators, it is difficult to draw conclusions on what makes an HCP creator reach a broad audience.

### Future Studies

Further studies should work to continue to characterize HCP content to gain an understanding of how HCPs can better combat misinformation. Examination of more than one hashtag could better categorize the growing field of vaccine-related content. Furthermore, comparing the TikToks at two different time points could better depict the ever-changing discourse of vaccine use on TikTok. Future studies should seek to understand the underlying causes that allow TikToks with blatant misinformation to succeed on the app.

### Conclusions

Given the 3 billion views of content about #covidvaccine [32], TikTok is clearly a platform where vaccine discourse is taking place. Although most of the content is provaccine, the smaller proportion of vaccine-hesitant content continues to receive more traffic in likes, shares, and comments, indicating that misinformation is still being engaged with on this platform. Encouragingly though, HCPs can play a role in curbing misinformation by posting provaccine content and establishing a larger presence on the app. Using TikTok and other social media responsibly is imperative to how health information will be spread around the globe. Studying these trends must be continued to understand how the world perceives medical information and how HCPs can improve trust in science and vaccines.

## Appendix

Multimedia Appendix 1 The most viewed TikToks with the hashtag #covidvaccine downloaded.

Multimedia Appendix 2 TikToks reviewed by 2 authors (JL and KvK) encoded or categorized.

## Abbreviations

HCP: health care professional
